# How well does neonatal neuroimaging correlate with neurodevelopmental outcomes in infants with hypoxic-ischemic encephalopathy?

**DOI:** 10.1038/s41390-023-02510-8

**Published:** 2023-03-01

**Authors:** Yvonne W. Wu, Sarah E. Monsell, Hannah C. Glass, Jessica L. Wisnowski, Amit M. Mathur, Robert C. McKinstry, Stefan Bluml, Fernando F. Gonzalez, Bryan A. Comstock, Patrick J. Heagerty, Sandra E. Juul

**Affiliations:** 1 Department of Neurology, University of California San Francisco, San Francisco, CA USA; 2 Department of Pediatrics, University of California San Francisco, San Francisco, CA USA; 3 Department of Biostatistics, University of Washington, Seattle, WA USA; 4 Department of Epidemiology, University of California San Francisco, San Francisco, CA USA; 5 Department of Radiology, Children’s Hospital Los Angeles, Los Angeles, CA USA; 6 Department of Pediatrics, Children’s Hospital Los Angeles, Los Angeles, CA USA; 7 Department of Pediatrics, Saint Louis University School of Medicine, St. Louis, MO USA; 8 Mallinckrodt Institute of Radiology, Washington Univ School of Medicine, St. Louis, MO USA; 9 Department of Radiology, University of Southern CA Keck School of Medicine, Los Angeles, CA USA; 10 Department of Pediatrics, University of Washington School of Medicine, Seattle, WA USA

## Abstract

**Background:**

In newborns with hypoxic-ischemic encephalopathy (HIE), the correlation between neonatal neuroimaging and the degree of neurodevelopmental impairment (NDI) is unclear.

**Methods:**

Infants with HIE enrolled in a randomized controlled trial underwent neonatal MRI/MR spectroscopy (MRS) using a harmonized protocol at 4–6 days of age. The severity of brain injury was measured with a validated scoring system. Using proportional odds regression, we calculated adjusted odds ratios (aOR) for the associations between MRI/MRS measures of injury and primary ordinal outcome (i.e., normal, mild NDI, moderate NDI, severe NDI, or death) at age 2 years.

**Results:**

Of 451 infants with MRI/MRS at a median age of 5 days (IQR 4.5–5.8), outcomes were normal (51%); mild (12%), moderate (14%), severe NDI (13%); or death (9%). MRI injury score (aOR 1.06, 95% CI 1.05, 1.07), severe brain injury (aOR 39.6, 95% CI 16.4, 95.6), and MRS lactate/n-acetylaspartate (NAA) ratio (aOR 1.6, 95% CI 1.4,1.8) were associated with worse primary outcomes. Infants with mild/moderate MRI brain injury had similar BSID-III cognitive, language, and motor scores as infants with no injury.

**Conclusion:**

In the absence of severe injury, brain MRI/MRS does not accurately discriminate the degree of NDI. Given diagnostic uncertainty, families need to be counseled regarding a range of possible neurodevelopmental outcomes.

**Impact:**

- Half of all infants with hypoxic-ischemic encephalopathy (HIE) enrolled in a large clinical trial either died or had neurodevelopmental impairment at age 2 years despite receiving therapeutic hypothermia.
- Severe brain injury and a global pattern of brain injury on MRI were both strongly associated with death or neurodevelopmental impairment.
- Infants with mild or moderate brain injury had similar mean BSID-III cognitive, language, and motor scores as infants with no brain injury on MRI.
- Given the prognostic uncertainty of brain MRI among infants with less severe degrees of brain injury, families should be counseled regarding a range of possible neurodevelopmental outcomes.

## Introduction

Hypoxic-ischemic encephalopathy (HIE), an important cause of neonatal encephalopathy, results from reduced oxygen and blood flow to a baby’s brain near the time of birth and is an important cause of long-term neurologic dysfunction.^1,2^ Survivors may demonstrate lifelong motor and cognitive deficits even after receiving hypothermia treatment.^3–8^ The extent of brain injury seen on neonatal brain MRI,^9–11^ and MR spectroscopy^12^ (MRS) has been shown to predict neurodevelopmental outcomes in infants who received therapeutic hypothermia for HIE with quantitative MRS measures providing better prognostic information than qualitative MRI scores of injury severity.^12,13^

Despite numerous studies of neuroimaging biomarkers of HIE, many questions remain. Although some studies found that a normal neonatal brain MRI is highly predictive of a normal outcome after HIE,^14,15^ other studies report a high (43%) rate of adverse neurodevelopmental outcomes despite a normal brain MRI.^16^ Furthermore, whether the acuity of brain lesions provides additional predictive value after HIE is unknown. Brain MRIs performed beyond 1 week of age may not distinguish acute from subacute lesions since the diffusion abnormalities that indicate acute injury “pseudonormalize” after 7 days.^17,18^

It is important to determine the best neuroimaging biomarkers of neurodevelopmental outcomes in infants with HIE, both to improve our ability to counsel families and to improve the design of future neuroprotection trials. Prior neuroimaging studies of HIE have been limited by the wide time window during which neuroimaging was performed,^11,14,16^ by small study size (<175 subjects),^9–11,14,16^ and by lack of harmonization of imaging protocols across sites.^14,16^ The HEAL Trial (NCT02811263) was a multi-center randomized controlled trial that tested erythropoietin in 500 infants who received therapeutic hypothermia for HIE. In this large cohort of infants who underwent a harmonized neuroimaging protocol within a narrow time window, we set out to determine the strength of association between MRI and MRS measures of brain injury and neurodevelopment at 2 years of age.

## Methods

### Overview

The HEAL study protocol^19^ and primary results^20^ have been previously published. Infants were eligible if they met all of the following criteria for presumed HIE: (1) born at ≥36 weeks’ gestation; (2) signs of perinatal depression (i.e., Apgar score < 5 at 10 min, cardiorespiratory resuscitation received beyond 10 min of age, or pH < 7.00 or base deficit ≥ 15 mmol/L in the cord or infant gas within 60 min of birth); (3) moderate or severe neonatal encephalopathy present at 1–6 h of age based on a modified Sarnat examination; and (4) underwent therapeutic hypothermia. The modified intention-to-treat analysis included 500 infants who were randomized and received five doses of Epogen 1000 U per kilogram or saline placebo intravenously during the first week of age. This study was approved by the Institutional Review Boards at all participating sites and neonates were studied after informed consent.

### Brain MRI

When possible, a clinical brain MRI was performed at 5 days (96–144 h) of age using a standardized protocol that was harmonized across nine different 3T MR scanners at 17 sites.^21^ The HEAL MRI protocol included conventional T1, T2, and diffusion-weighted sequences. Three independent readers reviewed the MRI images to determine the severity, pattern, and acuity of brain injury, with discrepancies resolved via consensus.

Using a validated scoring system,^22,23^ we calculated global brain injury scores by summing the extent of injury (i.e., none = 0; <25% = 1; 25–50% = 2; >50% = 3) seen on T1, T2, and apparent diffusion coefficient (ADC) images in eight regions of the brain: caudate, putamen/globus pallidus, thalamus, posterior limb of the internal capsule (PLIC), cortex, white matter, brainstem, and cerebellum. The severity of brain injury was determined from the global injury score as follows: none (global injury score = 0), mild (1–11), moderate (12–32), or severe (33–138) brain injury.

We defined three patterns of injury that were not mutually exclusive: *central gra*y, i.e., injury to the caudate, putamen, globus pallidus, or thalamus; *peripheral watershed*, i.e., injury to the parasagittal white matter or cortex; and *global*, i.e., injury to >75% of the cerebrum, consisting of central gray, white matter, and cortex. We further identified infants with *only punctate white matter lesions*, i.e., discrete 1–10 mm foci of injury in the periventricular white matter or centrum semiovale; and *only atypical lesions*, e.g., chronic volume loss, hemorrhage, or mass lesions.

Pseudonormalization of the ADC signal is expected to begin by 8 days after injury,^17^ and may not begin until 10–14 days in the setting of therapeutic hypothermia.^18^ Thus, we conservatively restricted the analysis of brain injury acuity to the 408 (91%) infants who received a brain MRI before 8 days (<193 h) of age. We defined *acute* lesions as foci of restricted diffusion; *subacute* lesions as signal abnormalities on T1 and/or T2 sequences but without corresponding diffusion restriction; and *chronic* lesions as parenchymal volume loss.^16,19^

### Proton MRS

Short-echo, single-voxel MRS data were acquired from the left thalamus and left parietal white matter. N-acetylaspartate (NAA) is a marker of neuronal/axonal integrity^23^ and lactate is a byproduct of anaerobic metabolism that increases in the setting of acute hypoxic-ischemic brain injury. We measured ratios of lactate/NAA and NAA/creatine as biomarkers of brain injury. Raw MRS data were processed centrally using a customized LCModel (V6.3-1L, Stephen Provencher Inc., Oakville, Ontario, Canada) pipeline as previously described.^16^ All spectra with major deviations in regions of interest (ROI) placement were excluded from subsequent analyses, as were any spectra with major artifacts (e.g., skull lipids, motion), poor linewidth (i.e., FWHM > 0.08) or signal-to-noise ratio <6.

### Neurodevelopmental impairment (NDI)

The primary outcome at 2 years (i.e., 22–36 months) of age was a five-level ordinal variable: no NDI, mild NDI, moderate NDI, severe NDI, or death. The severity of NDI was determined by the worst severity observed in either the cognitive or motor outcome. Cognitive outcome was defined by Bayley Scales of Infant Toddler Development, third edition (BSID-III) score as follows: normal (cognitive score ≥ 90); mild (85–89); moderate (70–84); severe (<70). Motor outcome (Table 1) was defined by the presence of cerebral palsy (CP) and by a modified Gross Motor Function Classification System^24^ (GMFCS) score (e-Fig. 1). Cerebral palsy was determined by a validated, standardized neurologic examination.^25^ Secondary outcomes included BSID-III cognitive, language, and motor scores among infants who survived to 2 years of age, and a binary outcome of ‘alive and no neurodevelopmental impairment’ vs. ‘died or some neurodevelopmental impairment.’

**Table 1:** Definition of motor outcome severity (i.e., normal, mild, moderate, severe) at age 2 years.

	Modified GMFCS score^a^						
**Cerebral palsy (CP)**	0	0.5	1	2	3	4	5
None	Normal	Normal	Mild	Moderate	Severe	Severe	Severe
Hemi- or Diparetic CP	Normal	Mild	Moderate	Moderate	Severe	Severe	Severe
Quadriparetic CP	Moderate	Moderate	Severe	Severe	Severe	Severe	Severe

^a^See Supplement for details of the Modified Gross Motor Function Classification scoring system.

### Statistics

The primary analysis compared the five-level ordinal outcome described above across MRI features by calculating adjusted odds ratios (aOR) using proportional odds regression.^26^ Two aORs are shown for each feature: one adjusting for treatment assignment (Epo vs. placebo) and site, and another adjusting for treatment assignment, site, and HIE severity. In analyses of the primary outcome, MRS ratios were standardized to rank order prior to modeling due to a high proportion of zero values among the lactate/NAA measurements. The secondary outcomes of BSID-III cognitive, language, and motor scores were compared using generalized linear models also adjusting for treatment, site, and HIE severity. Adjusted mean differences and corresponding 95% confidence intervals (CIs) are presented for each level of MRI injury compared to no injury. Those missing secondary outcomes due to death or attrition were excluded from secondary analyses, and the extent of missing data is reported in the text and tables. There were no missing data on treatment, site, HIE severity, or MRI severity, pattern, or acuity. To assess the predictive performance of MRI features, we estimated the sensitivity, specificity, negative predictive value, and positive predictive value of key MRI features in predicting the binary, secondary outcome of ‘alive and no neurodevelopmental impairment’ vs. ‘died or some neurodevelopmental impairment.’ Similarly, raw MRS ratios were evaluated for prediction using receiver operating characteristic (ROC) curves and associated area under the curve (AUC) analysis. All analyses evaluating predictive discrimination were unadjusted. Analyses were performed between August and October 2022 using R Statistical Software version 4.0.3 (R Foundation for Statistical Computing).

## Results

### Study cohort

Among 500 infants with presumed HIE who were participants in HEAL, the severity of encephalopathy was moderate in 77% and severe in 23%. A total of 474 (95%) infants received a brain MRI prior to discharge. The characteristics of infants with and without brain MRI were similar, except that infants without neuroimaging were more likely to have severe HIE (12/26, 46% vs. 101/474, 21%, *p* = 0.003) and to have died within 1 week of birth (20/26, 77% vs. 14/474, 3%, *p* < 0.001). We excluded four infants with uninterpretable scans due to excessive motion or administrative error, and 19 who had no primary outcome data available.

The final study cohort consisted of 451 infants who had an interpretable MRI study and a primary outcome at 2 years of age. The median age at neuroimaging was 5.0 days (IQR 4.5–5.8). Among infants in the study cohort, we assessed the acuity of brain injury in 408 (91%) infants who received an MRI prior to 8 days of age. We calculated MRS ratios in infants who had spectra of sufficient quality for analysis within the thalamus (*n* = 342, 76%) and white matter (*n* = 325, 72%).

About half (51%) of the study cohort had a normal neurodevelopmental outcome at age 2 years, while the remaining infants had mild (12%), moderate (14%), or severe (13%) NDI or died (9%). The median MRI brain injury score was 8 (IQR 2–22). The severity of the brain injury seen on MRI ranged from none (21%) to mild (37%), moderate (21%), and severe (21%) injury.

### Primary outcome

The MRI injury score (aOR 1.06, 95% CI 1.05, 1.07) and presence of severe brain injury compared to no injury (aOR 39.6, 95% CI 16.4, 95.6) were associated with worse outcomes as assessed by the primary outcome measure, both with and without adjusting for severity of encephalopathy (Table 2); in contrast, infants with mild or moderate brain injury had similar outcomes to those with no brain injury. The observed rate of death or any degree of NDI was 32/93 (34%) among infants with no MRI brain injury; 60/169 (36%) among infants with mild injury; 39/93 (42%) among infants with moderate injury; and 89/96 (93%) among infants with severe brain injury. Of the 42 infants who died, all had severe brain injury except for one child with moderate injury. Of 58 infants with severe NDI, 49 (85%) had either moderate or severe brain injury.

**Table 2:** Neonatal brain MRI and MRS findings and 2-year neurodevelopmental outcomes among 451 infants who received therapeutic hypothermia for HIE.

	All	No NDI	Mild NDI	Moderate NDI	Severe NDI	Death	OR (95% CI)^a^	aOR (95% CI)^b^
	*n* = *451*	*n* = *231*	*n* = *56*	*n* = *64*	*n* = *58*	*n* = *42*		
MRI injury score, median (IQR)	8 (2, 22)	4 (0, 12)	6 (0, 14.5)	6 (2, 14.25)	44 (15.25, 65.25)	81 (62, 110)	1.1 (1.1, 1.1)	1.1 (1.1, 1.1)
MRI injury severity								
None	93	61 (66)	15 (16)	13 (14)	4 (4)	0 (0)	Ref	Ref
Mild	169	109 (64)	23 (14)	32 (19)	5 (3)	0 (0)	1.2 (0.7, 2.2)	1.2 (0.7, 2.2)
Moderate	93	54 (58)	11 (12)	12 (13)	15 (16)	1 (1)	1.5 (0.8, 2.8)	1.4 (0.7, 2.6)
Severe	96	7 (7)	7 (7)	7 (7)	34 (35)	41 (43)	66.3 (28.1, 156.3)	39.6 (16.4, 95.6)
MRI patterns of injury								
No injury	139	94 (68)	21 (15)	20 (14)	4 (3)	0 (0)	Ref	Ref
Central gray	167	60 (36)	13 (8)	15 (9)	39 (23)	40 (24)	6.1 (3.7, 10.0)	4.3 (2.5, 7.3)
Peripheral watershed	111	45 (41)	13 (12)	14 (13)	27 (24)	12 (11)	4.4 (2.6, 7.6)	4.1 (2.3, 7.0)
Global	34	0 (0)	0 (0)	0 (0)	11 (32)	23 (68)	NA	NA
Only punctate white matter lesions	38	22 (58)	7 (18)	8 (21)	1 (3)	0 (0)	2.0 (0.9, 4.6)	2.1 (0.9, 4.9)
Only atypical pattern	29	16 (55)	4 (14)	5 (17)	3 (10)	1 (3)	2.1 (0.9, 5.4)	2.1 (0.8, 5.2)
MRI timing of injury	*n* = *408*	*n* = *212*	*n* = *49*	*n* = *54*	*n* = *52*	*n* = *41*		
No injury	125	87 (70)	17 (14)	17 (14)	4 (3)	0 (0)	Ref	Ref
Acute only	93	32 (34)	8 (9)	8 (9)	18 (19)	27 (29)	8.6 (4.6, 16.0)	6.7 (3.5, 12.7)
Subacute only	91	53 (58)	10 (11)	17 (19)	10 (11)	1 (1)	1.9 (1.0, 3.5)	1.9 (1.0, 3.6)
Acute + Subacute	90	36 (40)	12 (13)	10 (11)	19 (21)	13 (14)	5.2 (2.8, 9.7)	4.0 (2.1, 7.7)
Chronic (±acute/sub)	9	4 (44)	2 (22)	2 (22)	1 (11)	0 (0)	4.0 (0.9, 18.5)	3.8 (0.8, 17.7)
MRS thalamus, median (IQR)	*n* = *342*	*n* = *174*	*n* = *42*	*n* = *49*	*n* = *46*	*n* = *31*		
Lactate/NAA	0.07 (0.88, 0.16)	0.06 (0, 0.11)	0.07 (0.00, 0.13)	0.03 (0.00, 0.12)	0.20 (0.05, 0.45)	1.32 (0.52, 3.67)	1.7 (1.5, 1.9)^c^	1.6 (1.4, 1.8)^c^
NAA/Creatine	0.78 (0.69, 0.85)	0.81 (0.75, 0.89)	0.79 (0.69, 0.83)	0.79 (0.69, 0.83)	0.65 (0.60, 0.75)	0.62 (0.58, 0.68)	0.7 (0.7, 0.8)^c^	0.8 (0.7, 0.8)^c^
MRS white matter, median (IQR)	*n* = *325*	*n* = *160*	*n* = *42*	*n* = *47*	*n* = *45*	*n* = *31*		
Lactate/NAA	0.08 (0.00, 0.31)	0.04 (0.00, 0.22)	0.02 (0.00, 0.27)	0.03 (0.00, 0.20)	0.17 (0.00, 0.98)	0.89 (0.31, 2.86)	1.5 (1.3, 1.7)^c^	1.4 (1.2, 1.6)^c^
NAA/Creatine	0.78 (0.71, 0.89)	0.82 (0.75, 0.93)	0.79 (0.73, 0.86)	0.77 (0.69, 0.85)	0.74 (0.65, 0.80)	0.68 (0.58, 0.81)	0.8 (0.8, 0.9)^c^	0.8 (0.8, 0.9)^c^

NA not able to compute.

*n* (row percent) shown unless otherwise noted.

^a^Adjusted for treatment assignment and site. Note that treatment assignment was not associated with death or NDI in the main study analyses, as previously described.^20^

^b^Adjusted for treatment assignment, site, and severity of encephalopathy.

^c^In these models, spectroscopy scores were standardized to percentile; odds ratios are provided for a 10% increase in percentile.

Of 93 infants with no brain injury on MRI, none died and 61 (66%) had a normal outcome; the remaining infants included 15 (16%) with mild, 13 (14%) with moderate, and 4 (4%) with severe NDI. Four of the infants with no MRI evidence of brain injury but who nevertheless developed severe (*n* = 2) or moderate (*n* = 2) NDI were later diagnosed with an underlying genetic condition that accounted for their abnormal neurodevelopment.

Of 34 infants who had a global injury pattern, 23 (68%) died and the remaining 11 (32%) had severe NDI. Compared to infants with no brain injury, those with central gray (aOR 4.3, 95% CI 2.5–7.3) or peripheral watershed (aOR 4.1, 95% CI 2.3–7.0) also had a higher rate of adverse outcomes (Table 2). In contrast, the 38 (8%) infants with only punctate white matter lesions or 29 (6%) with only atypical lesions did not demonstrate a significantly increased risk of adverse outcomes when compared to infants with no brain injury.

Among 408 infants who received an MRI before 8 days of age, 283 (69%) had signs of brain injury; of these 283 infants, the injury was acute in 93 (33%), subacute in 91 (32%), and both acute and subacute in 90 (32%). Only nine (3%) infants had evidence of chronic injuries including volume loss in the white matter (6), deep gray nuclei (1), or cerebellum (1), and a remote germinal matrix hemorrhage (1). Compared to infants with no injury, those with acute injury alone (aOR 6.7, 95% CI 3.5–12.7), subacute injury alone (aOR 1.9, 95% CI 1.0–3.6), or both acute and subacute injury (aOR 4.0, 95% CI 2.1–7.7), were all more likely to have adverse outcomes (Table 2). Finally, both higher lactate/NAA ratios and lower NAA/creatine ratios were associated with worse outcomes, in both the thalamus and the parietal white matter (Table 2).

### Secondary outcomes

**Fig. 1:**
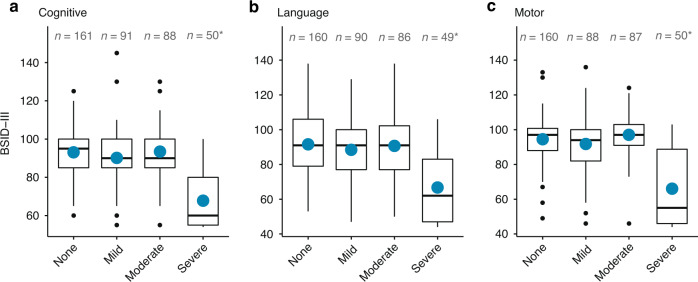
MRI severity of brain injury and Bayley Scales of Infant Development-III (BSID-III) outcomes. BSID-III **a** cognitive, **b** language, and **c** motor scores at 2 years of age in relation to the severity of MRI brain injury, among survivors of moderate to severe HIE. Blue dots represent mean value. *Indicates that the 95% CI for the mean difference does not contain 0. Mean difference (95% confidence interval), adjusted for site, treatment, and HIE severity as follows: cognitive: mild vs. none: -1.7 (-5.0,1.7); moderate vs. none: -1.4 (-3.6,0.9); and severe vs. none: -8.1 (-10.2, -6.0). Language: mild vs. none: -1.2 (-5.7,3.4); moderate vs. none: -1.6 (-4.5,1.3); and severe vs. none: -7.7 (-10.3, -5.1). motor: mild vs. none: -2.3 (-5.5,0.8); moderate vs. none: -1.9 (-4.1,0.3); and severe vs. none: -9.4 (-11.7,-7.1).

A total of 391 (87%) infants survived to the final endpoint and received a BSID-III evaluation at a median age of 24.7 months (IQR 23.9–25.9). Cognitive scores were available for 390 infants, while language and motor scores were available for 385 infants each. Compared to survivors with no injury on MRI, those with severe injury had significantly lower BSID-III cognitive (adjusted mean difference −8.1, 95% CI −10.2, −6.0), language (adjusted mean difference −7.7, 95% CI −10.3, −5.1), and motor (adjusted mean difference −9.4, 95% CI −11.7, −7.1) scores. In contrast, infants with mild or moderate injury did not exhibit significantly different BSID-III scores than infants with no injury (Fig. 1). Of note, even infants with no MRI injury had lower mean BSID-III scores than the general population (mean ± standard deviation: cognitive 93.5 ± 15.0; language 90.7 ± 19.4; motor 97.0 ± 11.9).

Central gray, peripheral watershed, and global injury patterns were each associated with worse BSID-III cognitive, language, and motor scores when compared to infants with no injury, while infants with only punctate white matter lesions or atypical lesions did not differ significantly in BSID-III scores when compared to infants with no injury (e-Fig. 2). Mean BSID-III scores did not vary significantly by the acuity of injury seen on MRI (e-Fig. 3).

**Fig. 2:**
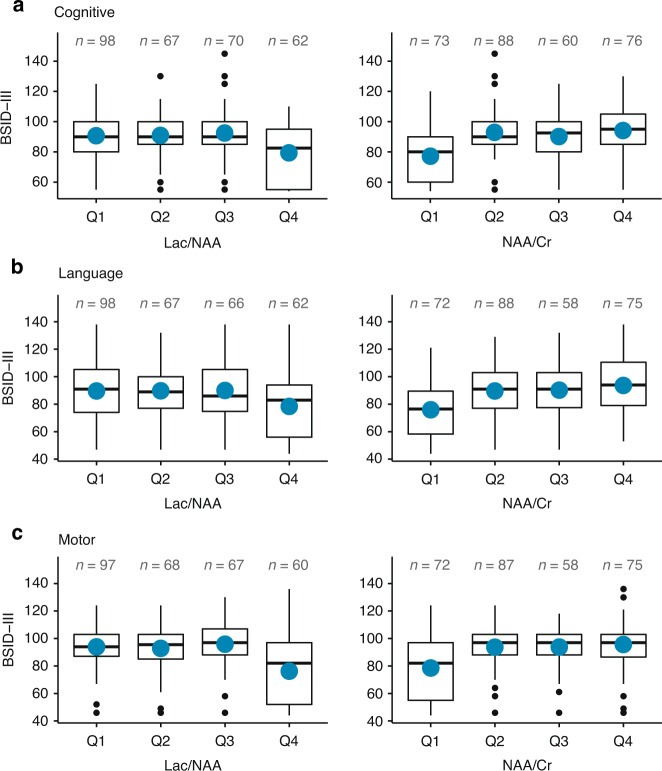
Thalamic MRS measures of brain injury and Bayley Scales of Infant Development-III (BSID-III) outcomes. BSID-III 10 **a** cognitive, **b** language, and **c** motor scores at 2 years of age in relation to lactate/NAA and NAA/Cr ratio quartiles in the thalamus among survivors of moderate to severe HIE. Mean change (95% confidence interval) in BSID-III score per 0.1 unit increase in lactate/NAA ratio, adjusted for site, treatment, and HIE severity: cognitive score: -0.7 (-1.1, -0.4); language score -0.8 (-1.3, -0.3); motor score -1.0 (-1.4, -0.5). Mean change (95% confidence interval) in BSID-III score per 0.1 unit increase in NAA/Cr ratio, adjusted for site, treatment, and HIE severity: cognitive score: 3.6 (1.9,5.3); language score 4.7 (2.6,6.8); motor score 4.4 (2.5,6.3).

Thalamic MRS data were available in 297 of 390 (76%) survivors who underwent a BSID-III evaluation. The thalamic lactate/NAA ratio was associated with all three BSID-III scores; i.e., for every 0.10 increase in this ratio, there was an adjusted mean difference of BSID-III scores as follows: cognitive −0.7, 95% CI −1.1, −0.4; language −0.8, 95% CI −1.3, −0.3; and motor −1.0, 95% CI −1.4, −0.5. The lower thalamic NAA/creatine ratio was similarly associated with lower BSID-III scores (Fig. 2), as were higher lactate/NAA and lower NAA/creatine ratios measured in the peripheral white matter (e-Fig. 4). The lactate/NAA ratio in both the thalamus and peripheral white matter was also associated with a higher risk of cerebral palsy (e-Table 1). The AUC for all MRS ratios for predicting the outcome of death or NDI ranged from 0.56 to 0.68 (e-Table 2).

**Fig. 3:**
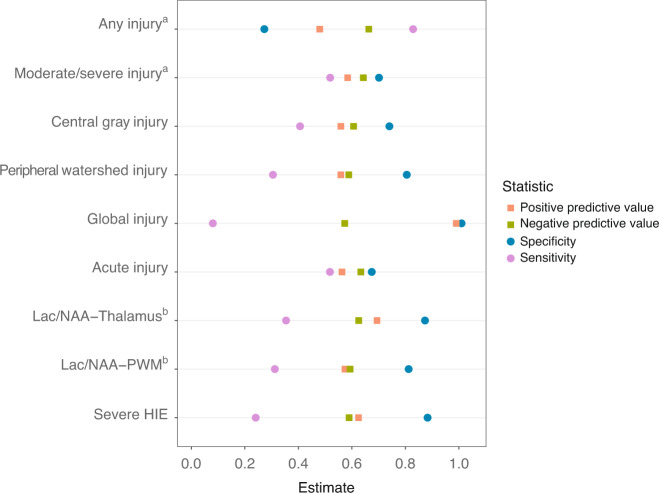
Diagnostic AQ11 accuracy of MRI, MRS, and clinical severity of HIE as predictors of death or NDI at 2 years of age, among infants with moderate to severe HIE. **a** Any injury is defined as MRI injury score >1; moderate/severe injury is defined as MRI injury score >11. **b** MRS measures are defined as top quartile of ratio vs. lower 3 quartiles of ratio values.

A global pattern of brain injury was the neuroimaging finding with the highest positive predictive value and highest specificity for predicting adverse 2-year outcomes, with all 34 affected infants either dying or developing severe NDI. The presence of any injury on MRI (i.e., global injury score > 0) had the highest sensitivity (0.83) for predicting the dichotomous outcome of death or NDI, while a lactate/NAA ratio in the top quartile in the thalamus (0.14 to 7.59) or peripheral white matter (0.27 to 5.13) demonstrated the highest specificity (0.87 and 0.81, respectively) for death or NDI (Fig. 3). The positive predictive values of all MRI and MRS features shown in Fig. 3 ranged from 0.48 to 1.00, while their negative predictive values ranged from 0.57 to 0.66. The presence of severe HIE on clinical examination had a positive predictive value of 0.63 and a negative predictive value of 0.59 for death or NDI.

## Discussion

In this large, prospective cohort of infants with HIE who underwent harmonized neuroimaging in the first week of age, we found several imaging biomarkers that were associated with 2-year neurodevelopmental outcomes including MRI severity of the injury, presence of central gray, peripheral white matter or global patterns of injury, and elevated lactate/NAA and lower NAA/creatine ratios on MRS. Although severe injury was strongly correlated with adverse outcomes, infants with mild or moderate brain injury exhibited rates of NDI and Bayley-III scores that were similar to infants with no apparent brain injury on MRI.

Clinicians frequently rely on brain MRI findings to counsel families on their infant’s future prognosis following HIE. Neonatal neuroimaging retains its predictive value even after infants have undergone therapeutic hypothermia, and MRS measurement of lactate/NAA in the thalamus has been shown to outperform MRI findings in predicting adverse outcomes after HIE.^12,13^ We found similarly that thalamic lactate/NAA ratio had a higher specificity and positive predictive value for an adverse outcome than any other neuroimaging feature, with the exception of the global injury pattern. However, the differences between MRI and MRS predictive values were small, and even the best MRS predictor (i.e., thalamic lactate/NAA) outperformed the clinical assessment of the severity of encephalopathy by only a small margin.

Like others,^16,27^ we found that neurodevelopmental impairment may occur in newborns with HIE who have no evidence of brain injury on neonatal MRI. There are several potential reasons for adverse neurodevelopmental outcomes in children with normal neuroimaging. First, HIE does not always occur in isolation, and the lack of HIE-related injury does not preclude a genetic abnormality that could predispose the infant to having both HIE and abnormal neurodevelopment. In our cohort, four subjects were later diagnosed with genetic abnormalities that could explain their abnormal neurodevelopmental outcomes despite an MRI that revealed no injury, and given the lack of systematic genetic testing, there may be additional genetic abnormalities that remain unrecognized in our subjects. Second, it is possible that some subjects will have abnormal NDI for reasons unrelated to HIE, such as traumatic or other brain injuries during infancy. Third, neurodevelopment is difficult to measure accurately at the age of 2 years, and mild delays seen on BSID-III and on neurologic examination can resolve as children continue to grow.^3,28^ Finally, although advanced neuroimaging techniques continue to improve our detection of HIE-related brain injury, subtle areas of injury may not always be detected due to insufficient resolution or residual movement artifacts.

Punctate white matter lesions were seen in 12% of infants enrolled in a large MRI study of healthy term newborns who had a normal exam and normal neurodevelopmental outcome.^29^ The same pattern of lesions have also been described in newborns with HIE^30,31^ but the prognostic implication of this finding had not been previously evaluated in a modern cohort of infants with HIE. In our study, the 38 infants with only punctate white matter lesions on brain MRI had neurodevelopmental outcomes that were no different from infants with no brain injury on MRI.

The ideal timing of brain MRI for predicting future neurodevelopment is unknown. Although some studies suggest an MRI performed in the first week of age may be most predictive,^32^ others found no difference between early and late neuroimaging in predicting outcomes.^33^ We report the first large-scale study of HIE that can distinguish acute from subacute injury because the majority (91%) of subjects received a brain MRI within the first week of age. We found that both acute and subacute injuries were associated with adverse outcomes in our cohort, with acute injury exhibiting the strongest association compared to no injury.

The fact that a static image of the brain obtained during the first week of age fails to accurately discriminate between milder forms of impairment is not surprising, as it does not measure the long trajectory of development that will continue to occur within the highly plastic newborn brain. It is well-known that maternal education is positively associated with better outcomes and that a disadvantaged socioeconomic status is associated with worse neurodevelopment after injury in both the term^3,7,34^ and preterm^35^ developing brain. Furthermore, early life exposures in the home environment are critical to childhood brain growth and cognitive development.^36,37^ How much the predictive value of neuroimaging findings would improve if combined with information regarding socioeconomic status and other HIE biomarkers such as clinical examination, EEG background, presence of seizures, and blood-based biomarkers of brain injury is beyond the scope of this study. Given the lack of certainty regarding the future neurodevelopmental trajectory of many infants with HIE, it is often important to describe a range of potential outcomes when counseling families. Because this prognostic uncertainty can be stressful, recent studies that incorporate feedback from family members are critically important in guiding clinicians on how best to approach these difficult discussions.^38–40^

Strengths of our study include the large sample size, prospective study design, harmonized neuroimaging protocol, short time window within which neuroimaging was performed, and the use of a validated MRI scoring system that could detect lesions in most regions of the brain.^41^ Similar to other detailed MRI scoring systems,^9^ our scoring system was able to detect a correlation between brain injury severity and all BSID-III sub-scores including language scores.^11^ Limitations of our study include the lack of long-term neurodevelopmental outcomes especially given the difficulty in accurately diagnosing mild NDI in a 2-year-old who may exhibit poor cooperation as opposed to true developmental delay; our inability to examine the predictive accuracy of early compared to late neuroimaging; and our lack of data regarding white matter microstructure^42^ or functional connectivity.^43^ Finally, although we assumed that infants in the subacute injury category experienced pseudonormalization of diffusion abnormalities prior to the acquisition of MRI, implying that injury occurred days prior to the time of birth, it is also possible that the diffusion was never abnormal and that some of the T1- and T2-weighted signal abnormalities in these infants represent edema as opposed to true injury.

In conclusion, several features on neonatal brain MRI and MRS were associated with worse neurodevelopmental outcomes after HIE. Although the finding of severe or global injury strongly predicts death or severe NDI, for infants with milder forms of brain injury, a range of outcomes are possible. Additional studies may improve prognostication by incorporating multiple modalities of biomarkers of brain injury.

## Supplementary information

Consort checklist

E-Table 1

Supplementary Information

Supp HEAL Consort Diagram

## Data Availability

We will prepare and share a final research data set that the accepted primary pragmatic trial publication is based upon. The final data set will be structured to maximize future scientific value while protecting patient and health system privacy. The UW DCC will remove or de-identify all HIPAA-specified direct identifiers. The aim of our data-sharing policy is to strive for the least restrictive plan possible while providing appropriate protection for participant privacy, health system privacy, and scientific integrity. Within 9 months of the end of the final year of funding (i.e., in the second half of 2023), a final study data set will be accessible via a supervised private data enclave managed by the National Institute of Neurological Disorder and Stroke (NINDS) at: https://www.ninds.nih.gov/Current-Research/Research-Funded-NINDS/Clinical-Research/Archived-Clinical-Research-Datasets. The shared data set will contain all data collected under both the HEAL Trial protocol and HEAL ancillary studies. Access will be limited to registered users who submit proposed specific questions or analysis plans and sign a data use agreement according to NINDS guidelines. “Supervised” indicates that individual requests are reviewed to protect the intellectual property rights of the project investigative team by restricting external development of manuscripts using the study data that substantially overlap with those that are already in development by study investigators.

## References

[1] Volpe JJ (2012). Neonatal encephalopathy: an inadequate term for hypoxic-ischemic encephalopathy. Ann. Neurol..

[2] Wu Y (2012). Brain injury in newborn babies: we can’t afford to get it wrong. Ann. Neurol..

[3] Pappas A (2015). Cognitive outcomes after neonatal encephalopathy. Pediatrics.

[4] Jary, S. et al. Motor performance and cognitive correlates in children cooled for neonatal encephalopathy without cerebral palsy at school age. *Acta Paediatr*. **108**, 1773–1780 (2019). 10.1111/apa.14780 30883895

[5] Azzopardi, D. Predictive value of the amplitude integrated EEG in infants with hypoxic ischaemic encephalopathy: data from a randomised trial of therapeutic hypothermia. *Arch. Dis. Child Fetal Neonatal Ed*. **99**, F80–F82. 10.1136/archdischild-2013-303710 PMC3888630 23800393

[6] Chalak, L. F. et al. Neurodevelopmental outcomes after hypothermia therapy in the era of Bayley-III. *J. Perinatol*. **34**, 629–633 (2014). 10.1038/jp.2014.67 PMC4117736 24743133

[7] Natarajan G (2014). Functional status at 18 months of age as a predictor of childhood disability after neonatal hypoxic-ischemic encephalopathy. Dev. Med. Child Neurol..

[8] Guillet R (2012). Seven- to eight-year follow-up of the CoolCap trial of head cooling for neonatal encephalopathy. Pediatr. Res..

[9] Weeke LC (2018). A novel magnetic resonance imaging score predicts neurodevelopmental outcome after perinatal asphyxia and therapeutic hypothermia. J. Pediatr..

[10] Bach AM (2021). Early magnetic resonance imaging predicts 30-month outcomes after therapeutic hypothermia for neonatal encephalopathy. J. Pediatr..

[11] Ni Bhroin M (2022). Relationship between MRI scoring systems and neurodevelopmental outcome at two years in infants with neonatal encephalopathy. Pediatr. Neurol..

[12] Lally PJ (2019). Magnetic resonance spectroscopy assessment of brain injury after moderate hypothermia in neonatal encephalopathy: a prospective multicentre cohort study. Lancet Neurol..

[13] Thayyil S (2010). Cerebral magnetic resonance biomarkers in neonatal encephalopathy: a meta-analysis. Pediatrics.

[14] Rutherford M (2010). Assessment of brain tissue injury after moderate hypothermia in neonates with hypoxic-ischaemic encephalopathy: a nested substudy of a randomised controlled trial. Lancet Neurol..

[15] Miller SP (2005). Patterns of brain injury in term neonatal encephalopathy. J. Pediatr..

[16] Shankaran S (2015). Neonatal magnetic resonance imaging pattern of brain injury as a biomarker of childhood outcomes following a trial of hypothermia for neonatal hypoxic-ischemic encephalopathy. J. Pediatr..

[17] McKinstry RC (2002). A prospective, longitudinal diffusion tensor imaging study of brain injury in newborns. Neurology.

[18] Bednarek N (2012). Impact of therapeutic hypothermia on MRI diffusion changes in neonatal encephalopathy. Neurology.

[19] Juul SE (2018). High-dose erythropoietin for asphyxia and encephalopathy (HEAL): a randomized controlled trial - background, aims, and study protocol. Neonatology.

[20] Wu YW (2022). Trial of erythropoietin for hypoxic-ischemic encephalopathy in newborns. N. Engl. J. Med..

[21] Wisnowski JL (2021). Integrating neuroimaging biomarkers into the multicentre, high-dose erythropoietin for asphyxia and encephalopathy (HEAL) trial: rationale, protocol and harmonisation. BMJ Open..

[22] Wu, Y. W. et al. High-dose erythropoietin and hypothermia for hypoxic-ischemic encephalopathy: a phase II trial. *Pediatrics* **137**, e20160191 (2016). 10.1542/peds.2016-0191 27244862

[23] Trivedi SB (2017). A validated clinical MRI injury scoring system in neonatal hypoxic-ischemic encephalopathy. Pediatr. Radiol..

[24] Palisano R (1997). Development and reliability of a system to classify gross motor function in children with cerebral palsy. Dev. Med. Child Neurol..

[25] Kuban KC (2008). An algorithm for identifying and classifying cerebral palsy in young children. J. Pediatr..

[26] McCullagh, P. Regression models for ordinal data. *J. R. Stat. Soc. Series B Methodol*. **42**, 109–142 (1980).

[27] van Kooij BJ (2010). Serial MRI and neurodevelopmental outcome in 9- to 10-year-old children with neonatal encephalopathy. J. Pediatr..

[28] Nelson KB, Ellenberg JH (1982). Children who “outgrew’ cerebral palsy. Pediatrics.

[29] Carney O (2021). Incidental findings on brain MR imaging of asymptomatic term neonates in the Developing Human Connectome Project. EClinicalMedicine.

[30] Hayman M (2019). Punctate white-matter lesions in the full-term newborn: underlying aetiology and outcome. Eur. J. Paediatr. Neurol..

[31] Li AM (2009). White matter injury in term newborns with neonatal encephalopathy. Pediatr. Res..

[32] O’Kane A (2021). Early versus late brain magnetic resonance imaging after neonatal hypoxic ischemic encephalopathy treated with therapeutic hypothermia. J. Pediatr..

[33] Rollins N (2014). Predictive value of neonatal MRI showing no or minor degrees of brain injury after hypothermia. Pediatr. Neurol..

[34] Miller SP (2002). Predictors of 30-month outcome after perinatal depression: role of proton MRS and socioeconomic factors. Pediatr. Res..

[35] Benavente-Fernandez I (2019). Association of socioeconomic status and brain injury with neurodevelopmental outcomes of very preterm children. JAMA Netw. Open..

[36] Hair NL, Hanson JL, Wolfe BL, Pollak SD (2015). Association of child poverty, brain development, and academic achievement. JAMA Pediatr..

[37] Hanson JL (2013). Family poverty affects the rate of human infant brain growth. PLoS ONE.

[38] Lemmon, M. E., Donohue, P. K., Parkinson, C., Northington, F. J. & Boss, R. D. Communication challenges in neonatal encephalopathy. *Pediatrics* **138**, e20161234 (2016). 10.1542/peds.2016-1234 PMC5005027 27489296

[39] Pilon B, Craig AK, Lemmon ME, Goeller A, Newborn Brain Society Guidelines Publications Committee (2021). Supporting families in their child’s journey with neonatal encephalopathy and therapeutic hypothermia. Semin. Fetal Neonatal Med..

[40] Cascio A (2022). Discussing brain magnetic resonance imaging results for neonates with hypoxic-ischemic encephalopathy treated with hypothermia: a challenge for clinicians and parents. eNeurologicalSci.

[41] Machie M (2021). MRI score ability to detect abnormalities in mild hypoxic-ischemic encephalopathy. Pediatr. Neurol..

[42] Bonifacio SL (2012). Therapeutic hypothermia for neonatal encephalopathy results in improved microstructure and metabolism in the deep gray nuclei. Am. J. Neuroradiol..

[43] Linke AC (2018). Disruption to functional networks in neonates with perinatal brain injury predicts motor skills at 8 months. Neuroimage Clin..

