# Supplementary material for: How well does neonatal neuroimaging correlate with neurodevelopmental outcomes in infants with hypoxic-ischemic encephalopathy?
Source: Pediatr Res. 2023 Mar 1;94(3):1018–25. doi: 10.1038/s41390-023-02510-8 (PMC10444609; doi:10.1038/s41390-023-02510-8)
Supplement: Supplementary file 1 — Consort checklist [file 41390_2023_2510_MOESM1_ESM.pdf]

CONSORT CHECKLIST

Please note that this article is a secondary analysis of data that were collected from a clinical trial.

Since the manuscript is not about the main findings of the trial (e.g., does not compare the two treatment groups), we believe that the consort checklist does not apply to this study.
