# Supplementary material for: How well does neonatal neuroimaging correlate with neurodevelopmental outcomes in infants with hypoxic-ischemic encephalopathy?
Source: Pediatr Res. 2023 Mar 1;94(3):1018–25. doi: 10.1038/s41390-023-02510-8 (PMC10444609; doi:10.1038/s41390-023-02510-8)
Supplement: Supplementary file 3 — Supplementary Information [file 41390_2023_2510_MOESM3_ESM.pdf]

e-Figure 1. Modified Gross Motor Function Classification System.

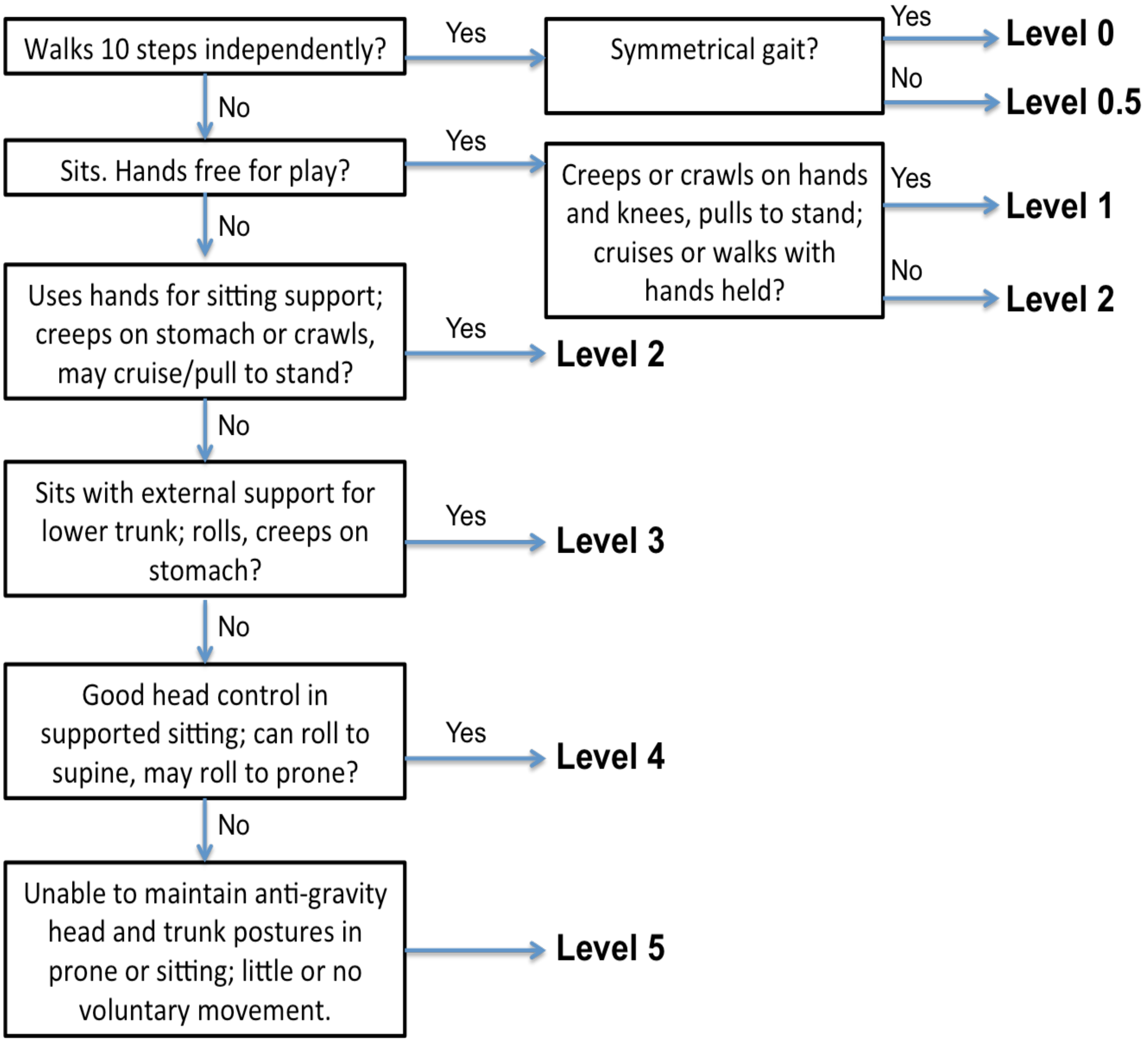

e-Figure 2. BSID-III cognitive, language and motor scores at 2 years of age in relation to pattern of MRI brain injury, among survivors of moderate to severe HIE.

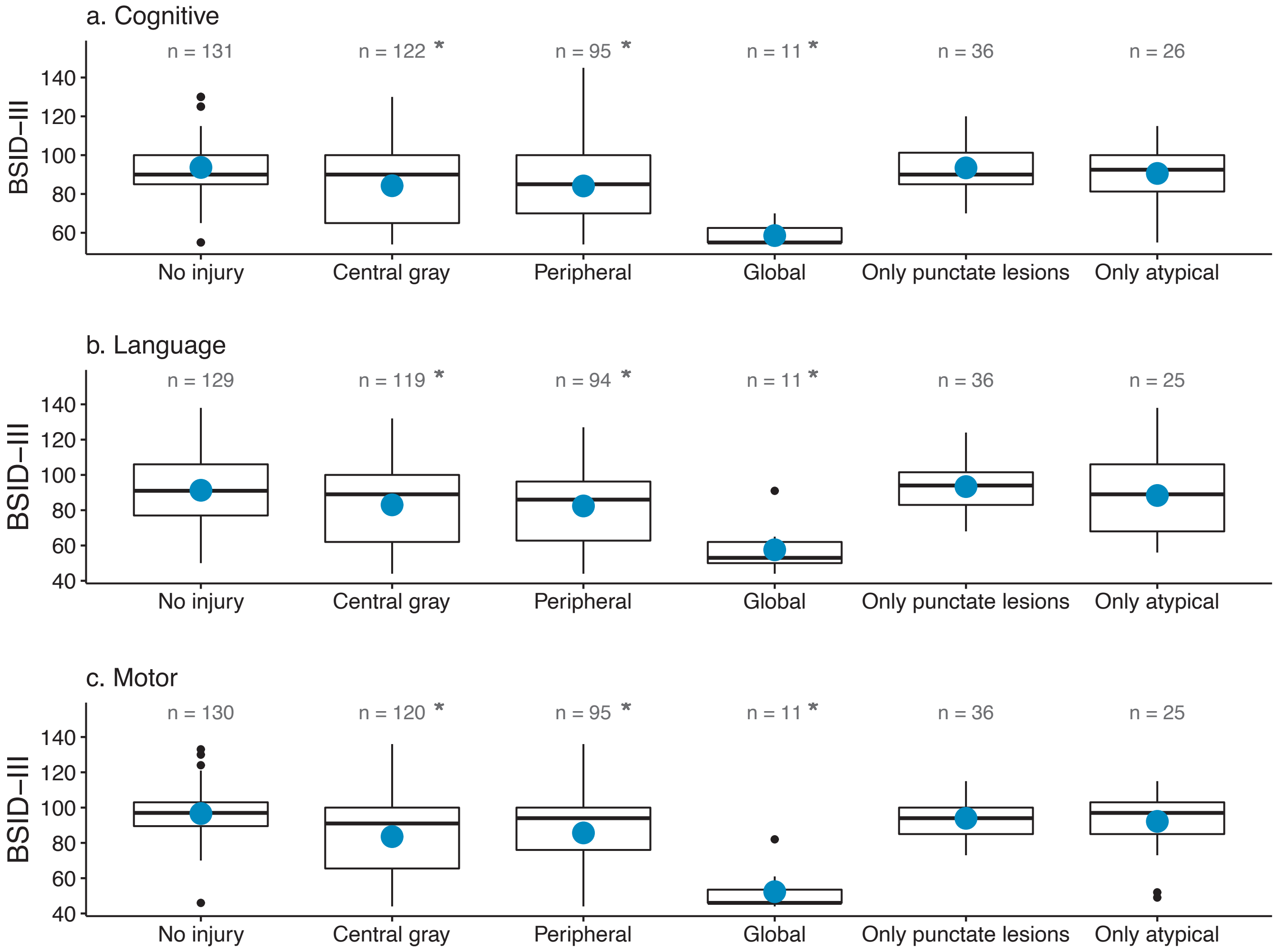

Blue dots represent mean value; *Indicates that the 95% CI for the mean difference does not contain 0. BSID-III cognitive: Central gray vs. No injury: -7.3 (-11.5,-3.1); Peripheral vs. No injury: -7.4 (-11.6,-3.2); Global vs. No injury: -35.7 (-44.1,-27.3); Only punctate lesions vs. No injury: -0.8 (-5.7,4.1); and Only atypical vs. No injury: -2.0 (-8.0,4.0). BSID-III language: Central gray vs. No injury: -6.7 (-12.0,-1.5); Peripheral vs. No injury: -7.3 (-12.5,-2.0); Global vs. No injury: -35.9 (-47.2,-24.6); Only punctate lesions vs. No injury: -1.7 (-8.2,4.7); and Only atypical vs. No injury: -1.3 (-9.3,6.7). BSID-III motor: Central gray vs. No injury: -10.0 (-14.7,-5.4); Peripheral vs. No injury: -8.7 (-13.3,-4.1); Global vs. No injury: -42.63 (-50.9,-34.3); Only punctate lesions vs. No injury: -3.1 (-7.5,1.4); and Only atypical vs. No injury: -1.8 (-7.6,4.1).

e-Figure 3. BSID-III cognitive, language and motor scores at 2 years of age in relation to lactate/NAA ratio quartiles in the parietal white matter among survivors of moderate to severe HIE.

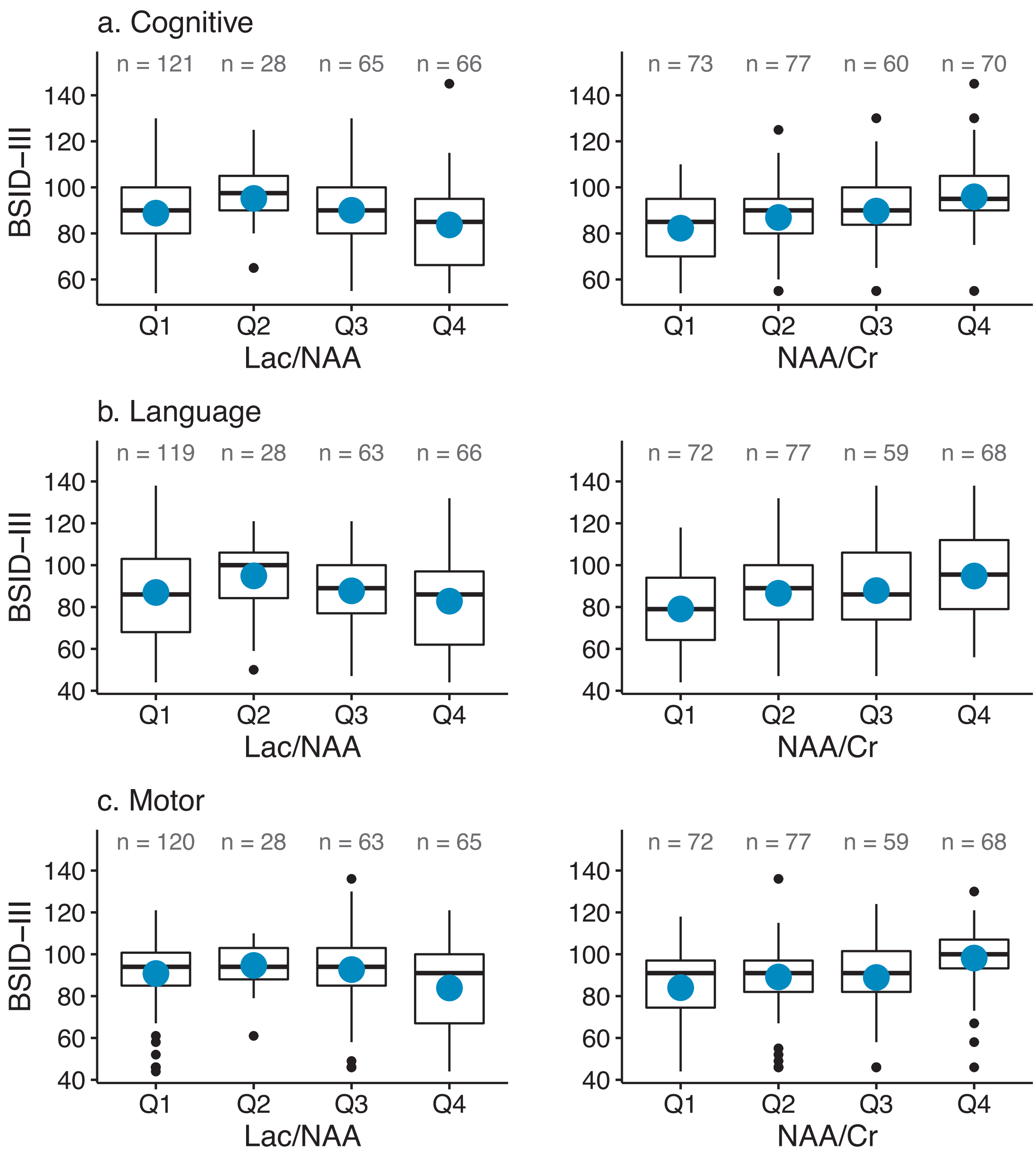

Mean increase (95% confidence interval) in BSID-III score per 0.1-unit increase in ratio, adjusted for site, treatment, and HIE severity are as follows. Lac/NAA: Cognitive: -0.9 (-1.3,-0.6); Language: -0.8 (-1.3,-0.4); Motor: -1.0 (-1.4,-0.6). NAA/Cr: Cognitive: 2.3 (1.0,3.5); Language: 2.9 (1.3,4.5); Motor: 3.0 (1.5,4.4).

e-Figure 4. BSID-III cognitive, language and motor scores at 2 years of age in relation to acuity of injury among survivors of moderate to severe HIE.

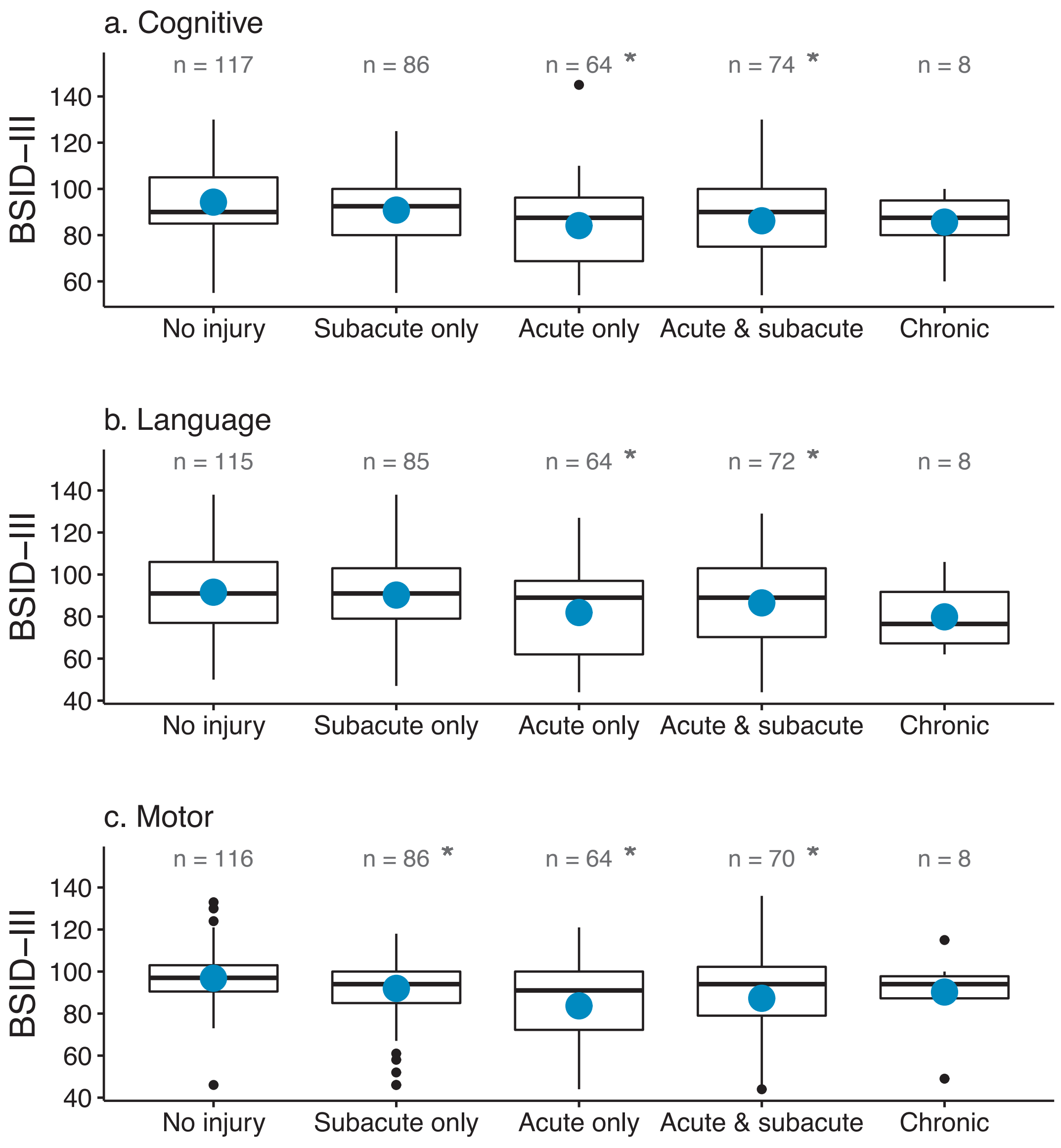

Note that the acuity is shown only for those with MRI <193 hours. Blue dots represent mean value; *Indicates that the 95% CI for the mean difference does not contain 0. BSID-III cognitive: Subacute only vs. No injury: -2.9 (-6.5,0.8); Acute only vs. No injury: -8.7 (-13.3,-4.2); Acute + subacute vs. No injury: -7.7 (-12.2,-3.2); and Chronic vs. No injury: -3.7 (-12.2,5.0). BSID-III language: Subacute only vs. No injury: -2.0 (-6.9,2.8); Acute only vs. No injury: -10.5 (-16.1,-4.9); Acute + subacute vs. No injury: -6.5 (-12.3,-0.7); and Chronic vs. No injury: -6.1 (-17.4,5.2). BSID-III motor: Subacute only vs. No injury: -4.2 (-7.6,-0.7); Acute only vs. No injury: -12.3 (-17.0,-7.6); Acute + subacute vs. No injury: -8.5 (-13.6,-3.4); and Chronic vs. No injury: -2.8 (-11.2,5.5).

e-Table 2. Among survivors of HIE, diagnostic accuracy of MRS measures for presence of death or NDI at age 2 years as measured by the area under the ROC curve (AUC).

	AUC (95% CI)*
**MR spectroscopy – thalamus.**	*n=318*
Lactate/NAA	0.60 (0.54-0.66)
NAA/Creatine	0.68 (0.62-0.74)
**MR spectroscopy - parietal white matter**	*n=301*
Lactate/NAA	0.56 (0.50-0.63)
NAA/Creatine	0.66 (0.60-0.72)

*95% CIs calculated using the method of DeLong

HEAL Consortium Members

Study Principal Investigators

Clinical Coordinating Center – Sandra E. Juul[1], Yvonne W. Wu[2]
Data Coordinating Center – Patrick J. Heagerty[1]

Site Principal Investigators

Kaashif A. Ahmad[3], Mariana Baserga[4], Ellen Bendel-Stenzel[5], Kristen L. Benninger[6], Lina Chalak[7], Taeun Chang[8], John Flibotte[9], Fernando F. Gonzalez[2], Andrea L. Lampland[10], Nathalie L. Maitre[11], Amit M. Mathur[12], Dennis E. Mayock[1], Stephanie Merhar[13], Ulrike Mietzsch[1,14], Brenda B. Poindexter[11], Rakesh Rao[15], David Riley[16], Christopher D. Smyser[15], Gregory M. Sokol[14], Krisa P. Van Meurs[17], Joern-Hendrik Weitkamp[18], Tai-Wei Wu[19], Toby D. Yanowitz[20]

Site Co-Investigators

Alexander G. Agthe[18], Nickie Andescavage[8], Sonia Bonifacio[17], Rachel Chapman[19], Kevin Dysart[9], Eric Eichenwald[9], Leah Engelstad[14], Donna M. Ferriero[2], Dawn Gano[2], Hannah C. Glass[2], Cody Henderson[3], Susan Hintz[17], Eugenia Ho[19], Natalia Isaza[8], Craig Jackson[1], Laura Jackson[20], Yvette Johnson[16], Russell Lawrence[16], Michelle Machie[7], Burhan Mahmood[20], An Massaro[8], Cristina Miller[10], Emily A. Morris[18], Hallie F. Morris[15], Monica Naik[20], Jonathan Nedrelow[16], Jamie R. Neumaier[18], Brighid O'Donnell[20], Elizabeth E. Rogers[2], Pablo J. Sanchez[6], Jennifer Shepherd[19], Jennifer Thomas[7], Tammy Tsuchida[8], Zachary Vesoulis[15], Vivek Vijayamadhavan[3], Sarah Winter[4]

Site Follow-Up Personnel

Kate Bataglia[10], Nancy Batterson[6], Brooke Bernstein[6], Suzin Blankenship[15], Sonia Bonifacio[17], Fiona Burrows[3], Lauren Christopher[2], Arturo Corrales[16], Sean Cunningham[4], Jo Ann D'Agostino[9], Maria-Elena DeAnda[17], Anne DeBattista[17], Sara B. Demauro[9], Gina Drury[13], Andrea F. Duncan[9], Nathalie ElTers[15], Cathy Neis Farrell[10], Mario Fierro[3], Nancy Forero[19], Charlotte Frey[3], Karen Blanc Friedman[9], Susan Friedman[9], Janell Fuller[21], Thomas George[10], Marsha Gerdes[9], April German[13], Kendell German[1], Shirley Gogliotti[18], Megan Goldenshteyn[1], Rachel H. Goode[18], Mary Haman[15], Candace Hawthorne[20], Anne Hay[1], Yvonne Hayden[14], Roy Heyne[7], Abbey Hines[14], Casey Hoffman[9], Betty Hutchon[22], Erika Jensen[4], Bridget Johnson[2], Yvette Johnson[16], Katelyn Keener[6], Aimee Kramer[15], Casey Krueger[17], Alice Lawrence[18], Marsha Lee[13], Kathleen Lehman[1], Melissa Liebowitz[2], Melissa Liggett[8], Lauren Lorenzi-Quigley[20], Pamela Lundequam[10], Elsa Malmud[9], Stephanie McCall[6], Kelly McHugh[13], Cristina Miller[10], Hallie F. Morris[15], Galina Morshedzadeh[4], Amanda L. Mouvery[18], Emily Myers[1], Monica Naik[20], Nina Natarajan[1], Mary Lauren Neel[6], Mary Ann Nelin[6], Cari Oakes[13], Jessica Patel[17], Lindsay Pietruszewski[6], Erin Plummer[10], Deborah A. Powers[18], Rebecca Rapoport[2], Hisela Rauda[19], Liz Rick[13], Lauren Scott[10], Elizabeth Siqveland[10], Laurel A. Slaughter[6], Lacey Stonebraker[14], Kelly Stout[4], Ashley Stuart[4], Heather Taylor[17], Kristine Tolentino-Plata[7], Tammy Tsuchida[8], Douglas Vanderbilt[19], Justine Vecchiarelli[20], Zachary Vesoulis[15], Angela N. Vierling[15], Natalie Wager[17], Donna Watkins[14], Sarah Wing[14], Sarah Winter[4], Cortney Wolfe-Christensen[16], Elizabeth Zorn[10]

Site Clinical Research Coordinators

Victoria Autelli[19], Shawna Baker[4], Bethany Ball[17], Anthony Barton[15], Kelsey Bassett[16], Laura Cole Bledsoe[4], Frannie Boyle[14], Bailey Clopp[1], Kylie Corry[1*], Melanie Drummond[3], Juanita Dudley[13], Aaron Espinoza[3], Isabella Esposito[1], John Feltner[1†], Dana Fine[19], Molly Fisher[10], Alexis Gossett[16], Cathy Grisby[13], Stephanie Hauge*[1], Kinnedy Houston[19], Brian Kaletka[10], Stacey Kleinman[9], Katy Kohlleppel[3], Lizette Lee[7], Penny Li[8], Meaghan McGowan[8], Kelleen Nelson[2‡], Samantha Nikirk[1], Alexandra O'Kane[8], Tira Oskoui[8], Jessica Purnell[6], Hannah Rakow[14], Carrie Rau[4], Elizabeth Reichert[17], Theresa Rogers[18], Emmeline Roth[8], Pollieanna Sepulveda[7], Amy Silvia[1], Sara Stacey[13], Emily Strait[14], Dawn Thomas[20], Brittany Toda-Eng[19], Danielle D. Weinberg[9], Sarah Wells[16], Ashley Widmayer[6], Cathy Worwa[10], Sandra Wuertz[13], Ashlyn Yarnell[16], Kristina Ziolkowski[9]

**Biomarkers Central Coordinator*
†Lead Central Coordinator
‡Neuroimaging Central Coordinator

University of Washington Data Coordinating Center

Bryan A. Comstock[1], Patrick J. Heagerty[1], Mark A. Konodi[1], Christopher Nefcy[1]

HEAL Executive Committee
Roberta Ballard[2], Bryan A. Comstock[1], John Feltner[1], Donna M. Ferriero[2], Fernando F. Gonzalez[2], Amy M. Goodman[2], Patrick J. Heagerty[1], Sandra E. Juul[1], Dennis E. Mayock[1], Elizabeth E. Rogers[2], Yvonne W. Wu[2]

HEAL Project Director
Amy M. Goodman[2]

HEAL Medical Monitor
Michael D. Schreiber[23]

HEAL Follow-Up Committee
Karl C. K. Kuban[24], Jean R. Lowe[21], T. Michael O'Shea[25], Elizabeth E. Rogers[2*], Yvonne W. Wu[2]
**Follow-Up PI*

HEAL Neuroimaging Committee
Stefan Bluml[19], Amit M. Mathur[12], Robert C. McKinstry[15*], Ashok Panigrahy[20], Jessica L. Wisnowski[19*], Yvonne W. Wu[2]
**Neuroimaging PIs*

HEAL Biomarkers Committee
Theo Bammler[1], Sandra E. Juul[1], An Massaro[8*], Adam L. Numis[2*], Yvonne W. Wu[2]
**Biomarker PIs*

Affiliations

1. University of Washington (Seattle, WA)
2. University of California, San Francisco (San Francisco, CA)
3. Pediatrix Medical Group of San Antonio (San Antonio, TX)
4. University of Utah (Salt Lake City, UT)
5. Mayo Clinic (Rochester, MN)
6. Nationwide Children’s Hospital (Columbus, OH)
7. University of Texas Southwestern Medical Center (Dallas, TX)
8. Children’s National Hospital (Washington, DC)
9. Children’s Hospital of Philadelphia (Philadelphia, PA)
10. Children’s Minnesota (Minneapolis/St. Paul, MN)
11. Children’s Healthcare of Atlanta and Emory University (Atlanta, GA)
12. Saint Louis University School of Medicine (St. Louis, MO)
13. Cincinnati Children’s Hospital (Cincinnati, OH)
14. Indiana University School of Medicine (Indianapolis, IN)
15. Washington University in St. Louis (St. Louis, MO)
16. Cook Children’s Medical Center (Ft. Worth, TX)
17. Stanford University (Stanford, CA)
18. Vanderbilt University Medical Center (Nashville, TN)
19. Children’s Hospital Los Angeles (Los Angeles, CA)
20. University of Pittsburgh School of Medicine & Children’s Hospital of Pittsburgh of UPMC and Magee (Pittsburgh, PA)
21. University of New Mexico School of Medicine (Albuquerque, NM)
22. Royal Free London NHS Trust (London, UK)
23. The University of Chicago (Chicago, IL)
24. Boston University Medical Center (Boston, MA)
25. University of North Carolina (Chapel Hill, NC)
