# Supplementary figures and images for: How well does neonatal neuroimaging correlate with neurodevelopmental outcomes in infants with hypoxic-ischemic encephalopathy?
Source: Pediatr Res. 2023 Mar 1;94(3):1018–25. doi: 10.1038/s41390-023-02510-8 (PMC10444609; doi:10.1038/s41390-023-02510-8)

## HEAL Consort Diagram

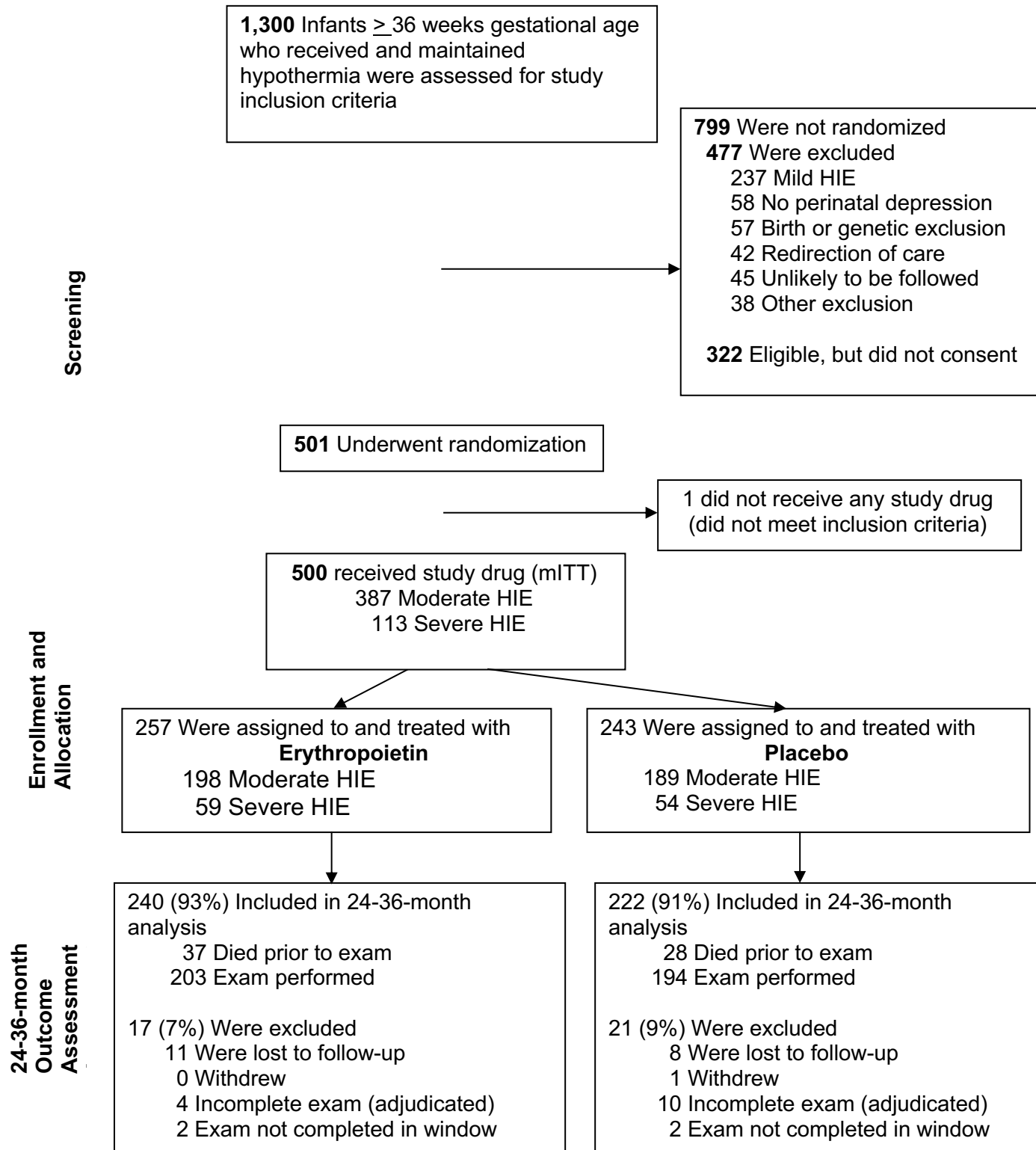

Supplement: Supplementary file 4 — Supp HEAL Consort Diagram [file 41390_2023_2510_MOESM4_ESM.pdf]
